# Clinical decision support analysis of a microRNA-based thyroid molecular classifier: A real-world, prospective and multicentre validation study

**DOI:** 10.1016/j.ebiom.2022.104137

**Published:** 2022-07-01

**Authors:** Marcos Tadeu Santos, Bruna Moretto Rodrigues, Satye Shizukuda, Andrei Félix Oliveira, Miriane Oliveira, David Livingstone Alves Figueiredo, Giulianno Molina Melo, Rubens Adão Silva, Claudio Fainstein, Gerson Felisbino dos Reis, Rossana Corbo, Helton Estrela Ramos, Cléber Pinto Camacho, Fernanda Vaisman, Mário Vaisman

**Affiliations:** a Research and Development (R&D), Onkos Molecular Diagnostics, Ribeirão Preto, SP, Brazil; b Molecular Oncology Research Centre, Barretos Cancer Hospital, Barretos, SP, Brazil; c Head and Neck, Midwestern State University (UNICENTRO), Guarapuava, PR, Brazil; d Otorhinolaryngology, Head and Neck Surgery, Paulista Medical School/UNIFESP, São Paulo, SP, Brazil; e Head and Neck Surgery, The Portuguese Beneficence of São Paulo (BP), São Paulo, SP, Brazil; f Surgical Cancerology, Complexo ISPON, Ponta Grossa, PR, Brazil; g General Surgery, Fluminense Federal University (UFF), Niterói, RJ, Brazil; h Head and Neck Surgery, University of São Paulo (USP) HC-FMRP, Ribeirão Preto, SP, Brazil; i Endocrinology, National Cancer Institute (INCA), Rio de Janeiro, RJ, Brazil; j Health and Sciences, Federal University of Bahia (UFBA), Salvador, BA, Brazil; k Endocrinology, Paulista Medical School/UNIFESP, São Paulo, SP, Brazil; l Endocrinology, Medical School, Rio de Janeiro Federal University (UFRJ), Rio de Janeiro, RJ, Brazil

**Keywords:** MicroRNA, Indeterminate thyroid nodule, Thyroid cancer, Molecular diagnostics, Molecular classifier, Real-world evidence

## Abstract

**Background:**

The diagnosis of cancer in Bethesda III/IV thyroid nodules is challenging as fine-needle aspiration (FNA) has limitations, and these cases usually require diagnostic surgery. As approximately 77% of these nodules are not malignant, a diagnostic test accurately identifying benign thyroid nodules can reduce “potentially unnecessary” surgery rates. We have previously reported the development and validation of a microRNA-based thyroid classifier (mir-THYpe) with high sensitivity and specificity, which could be performed directly from FNA smear slides. We sought to evaluate the performance of this test in real-world clinical routine to support clinical decisions and to reduce surgery rates.

**Methods:**

We designed a real-world, prospective, multicentre study. Molecular tests were performed with FNA samples prepared at 128 cytopathology laboratories. Patients were followed-up from March 2018 until surgery or until March 2020 (patients with no indication for surgery). The final diagnosis of thyroid tissue samples was retrieved from postsurgical anatomopathological reports.

**Findings:**

A total of 435 patients (440 nodules) classified as Bethesda III/IV were followed-up. The rate of avoided surgeries was 52·5% for all surgeries and 74·6% for “potentially unnecessary” surgeries. The test achieved 89·3% sensitivity, 81·65% specificity, 66·2% positive predictive value, and 95% negative predictive value. The test supported 92·3% of clinical decisions.

**Interpretation:**

The reported data demonstrate that the use of the microRNA-based classifier in the real-world can reduce the rate of thyroid surgeries with robust performance and support clinical decision-making.

**Funding:**

The São Paulo Research-Foundation (FAPESP) and Onkos.

**Research in context**

**Evidence before this study**

We have previously reported the development and validation of a microRNA-based molecular classifier (mir-THYpe) for thyroid nodules with indeterminate cytology with high sensitivity and specificity, which can be performed directly from readily available cytological smear slides without the need for a new dedicated FNA and at a significantly lower cost. Despite the high performance observed in this retrospective study, a prospective approach assessing the test performance in the real clinical practice was needed to validate its true potential to reduce thyroid surgeries and support clinical decisions. We performed a systematic search for studies (by title and abstract) evaluating the performance or the clinical utility of thyroid molecular diagnostic tests that classify indeterminate nodules and included articles or conference abstracts published from January 1, 2000 to January 31, 2022, in English, Portuguese, or Spanish. We used the PubMed and the Scientific Electronic Library Online (SciELO) for articles and world conferences of the American Thyroid Association (ATA), European Thyroid Association (ETA), Latin American Thyroid Society (LATS), and World Congress on Thyroid Cancer (WCTC) for abstracts/posters. Search terms included (with or without the Boolean AND) “thyroid nodule,” “thyroid cancer,” “indeterminate thyroid nodule,” “molecular classifiers,” “gene-expression,” “miRNA OR microRNA,” “DNA mutation(s),” “expression profile,” “real-world,” “clinical utility,” and “clinical validation.” Next, we filtered the validation studies on molecular classifier tests that are currently available for commercial use in the clinical setting published by the providers. We were not able to identify a real-world, prospective, multicentre study on microRNA-based thyroid molecular classifiers, highlighting the clear need for a study evaluating their real impact on supporting clinical decisions and reducing thyroid surgery rates.

**Added value of this study**

In this real-world evidence, prospective, multicentre study, 435 patients with 440 Bethesda III/IV nodules were tested and followed-up. Clinical samples (FNA smear slides) used for molecular testing were prepared at 128 cytopathology laboratories. The rate of avoided surgeries was 52·5% for all surgeries and 74·6% for “potentially unnecessary” surgeries. The mir-THYpe test achieved 89·3% sensitivity (CI95% 82–94·3), 81·65% specificity (CI95% 76·6–86), 66·2% positive predictive value (CI95% 60·3–71·7), and 95% negative predictive value (CI95% 91·7–97) at 28·7% (CI95% 24·3–33·5) cancer prevalence. The test supported 92·3% of clinical decisions.

**Implications of all the available evidence**

The reported data demonstrate that the use of a microRNA-based classifier in the real-world can reduce the rates of thyroid surgeries with robust performance comparable to other well-established thyroid molecular classifiers and support clinical decision-making. Cost-effectiveness analyses of the test's financial impact and healthcare system savings are under development, and, together with the present data, will help guide new policies.

Alt-text: Unlabelled box

## Introduction

Thyroid cancer has increased in the last few decades, and it ranks as the ninth most-incident cancer worldwide.^1^ Although there is consensus that overdiagnosis is the main reason for increased incidence^2^^,^^3^ mainly due to incidental thyroid nodules found on imaging screenings performed for reasons other than thyroid disease evaluation,^4^ there has also been a true increase in the occurrence of thyroid cancer.^5^

Fine-needle aspiration (FNA) cytology is the current gold standard for triaging patients with suspicious thyroid nodules detected clinically or on ultrasound (US),^6^, ^7^, ^8^ and the six-tier Bethesda System for Reporting Thyroid Cytopathology attempts to standardise cytopathologic analysis.^9^

Approximately 64% of histologically diagnosed thyroid cancer nodules were initially classified on FNA cytology as Bethesda V or VI, with a combined risk of malignancy (RoM) of 91% (only 1·1 surgeries required to detect one case of cancer).^10^ Although the proportion of histologically diagnosed thyroid cancer nodules initially classified on FNA cytology as “indeterminate” (Bethesda III and IV) is lower (∼29%), the combined RoM for these two classes is about 23% only,^10^ implying that around 77% of surgeries are “potentially unnecessary” and could have been reconsidered or avoided (4·4 surgeries required to detect one case of cancer).

Molecular testing has emerged as an option to reduce the need for diagnostic surgery in indeterminate thyroid nodules,^8^ being recommended by the American Thyroid Association (ATA)^11^ and the National Comprehensive Cancer Network (NCCN)^12^ guidelines. We have recently reported the development and validation of a new microRNA-based thyroid molecular classifier test for a precision endocrinology (mir-THYpe) with high sensitivity and specificity, which could be performed directly from readily available cytological smear slides at a significantly lower cost, without the need for a new dedicated FNA.^13^^,^^14^

We present data of a real-world, prospective, and multicentre study to evaluate the support of mir-THYpe results on clinical decisions and on reducing surgery rates in real-world clinical settings and to analyse its performance on Bethesda III and IV nodules.

## Methods

### Study design and patient data

As we conducted a real-world study to prospect evidence of the utility and performance of the test, patients eligible for this study were consecutive and had a signed medical prescription for the use of a mir-THYpe test performed in clinical routine with at least one thyroid nodule previously biopsied by FNA and classified as Bethesda III or IV. Ultrasound features and cytological analysis were just considered by the physicians who prescribed the test. We did not interfere with patient selection or exclusion. After molecular test results, patients were assigned to one of the two groups: Group A underwent thyroidectomy (tests performed March/2018–March/2020); Group B did not undergo thyroid surgery (tests performed March/2018–October/2019, follow-up in March/2020). Patients initially assigned to Group B subsequently undergoing thyroidectomy were included in Group A.

Patients who underwent thyroid surgery but for whom we could not obtain original postsurgical anatomopathological (AP) reports were excluded. Samples in which the FNA cytology report and other characteristics, such as size and location, did not correlate with the nodule described in the AP report were also excluded from the performance analysis.

### Ethics

Patients were 18 years or older, provided written informed consent, and paid out-of-pocket for the test. The study was approved by the institution's Research Ethics Committee and listed under CAAE number 39107520.0.0000.0106.

### Samples

**Figure 1:**
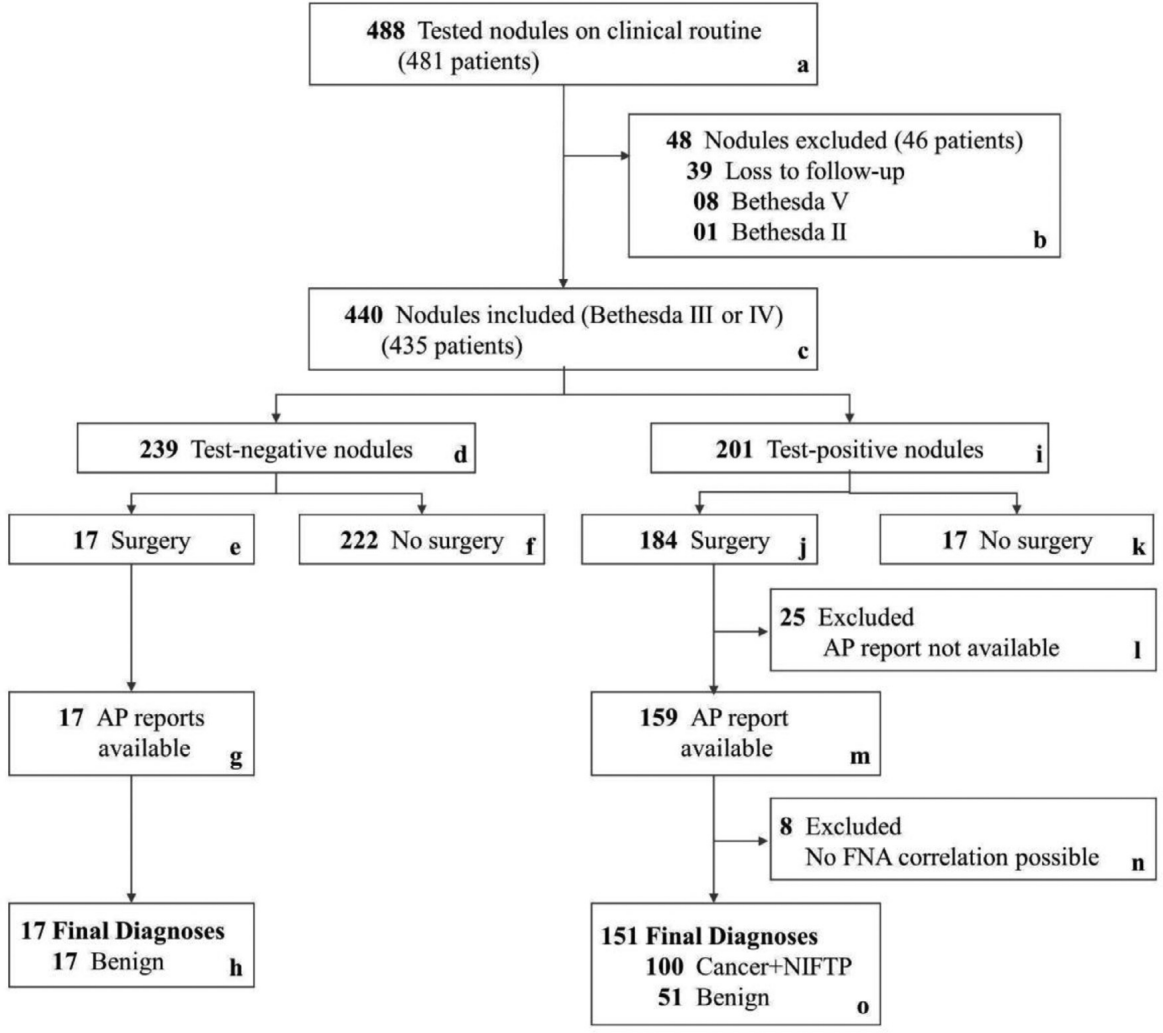
**Patients and Nodules Exclusion Flow Chart.** AP, anatomopathological; FNA, fine-needle aspiration; NIFTP, non-invasive follicular thyroid neoplasm with papillary-like nuclear features.

Other pathologists did not revise the FNA cytology Bethesda classes and the postsurgical histological classification of resected nodules. The FNA smear slides of the 440 samples (used for the mir-THYpe test – Figure 1, box c) were prepared, and the Bethesda classes were assigned by 128 cytopathology laboratories (Supplementary Figure S1). All fixation and staining protocols of the FNA cytology smear slides are eligible for the mir-THYpe test. The samples were sent without temperature control (room-temperature). Thyroidectomy AP reports were used as reference standard (gold-standard), when available. The 168 postsurgical AP reports with the histological classification of nodules (Figure 1, boxes h and o) were assigned by 53 pathology laboratories. Although the benign/indolent behaviour of the non-invasive follicular thyroid neoplasms with papillary-like nuclear features (NIFTPs),^15^ its diagnosis is established only after applying post-surgery stringent histological criteria.^14^ Therefore, we consider NIFTPs as “malignant” tissues and positive tests were considered true positives.

### Molecular analysis

The mir-THYpe molecular classifier test was performed according to the previously described method.^13^ A 12^th^ microRNA (miR-375) was also analysed to screen for medullary thyroid cancer.^16^ No mutation or DNA analyses were performed on the samples. Due to its prospective nature, the present study could only be compared with other prospective studies performed by commercial test suppliers.

### Statistics

Statistical analyses were performed using the R software, an open-source statistical programming environment. The confidence intervals for sensitivity, specificity, and accuracy were the “exact” Clopper–Pearson confidence intervals, and the confidence intervals for the predictive values were the standard logit confidence intervals.^17^ Confidence intervals for the means were calculated using the method described by Walline.^18^ The Bayes’ theorem used to calculate the negative predictive value (NPV) of non-resected nodules was based on Hall.^19^ Consecutive unblinded sampling without randomization was applied in order to meet the inclusion/exclusion criteria described on “Study design and patient data” and the real-world and prospective design aimed for this study.

### Role of funders

The funder (Onkos) designed the study with assistance from academic researchers. The funder (Onkos) aided in data collection, analysis, and interpretation, and in report writing. More than one author had full access to all the data from the study and verified the data reported in the manuscript. The corresponding author had final responsibility for the decision to submit for publication and the authors were not precluded from accessing data in the study and accepted responsibility for submitting for publication.

## Results

### Patient and nodule characteristics

A total of 481 real-world patients with 488 nodules (1·01 nodule/patient) and a medical prescription for the mir-THYpe test provided written informed consent. Of these, 39 nodules (8%) were excluded due to loss to follow-up. Eight nodules (1·6%) classified as Bethesda V and one nodule (0·2%) classified as Bethesda II were also excluded (Figure 1, box b) (only Bethesda III/IV nodules were accepted for this study). The remaining 440 nodules classified as Bethesda III and IV, from 435 patients (1·01 nodules/patient), which fulfilled all initial inclusion criteria described in study design and patient data, were categorised according to the mir-THYpe test results (Figure 1 and Table 1).

**Table 1:** Demographic and clinical characteristics of the study cohort.

		mir-THYpe test result	
*Variable*	Total	Negative	Positive
**Cohort, No (%)**			
Nodules Bethesda III/IV	440	239 (54·3)	201 (45·7)
Unique Patients^a^	435	-	-
**Sex, No (%)**			
Male	79 (18)	50 (63·3)	29 (36·7)
Female	361 (82)	189 (52·4)	172 (47·6)
**Age, No (%), y**			
20-54	252 (57·3)	124 (49·2)	128 (50·8)
≥ 55-90	188 (42·7)	115 (61·2)	73 (38·8)
**Bethesda Classes**^b^**, No (%)**			
AUS/FLUS (III)	247 (56·1)	126 (51)	121 (49)
FN/SFN (IV)	193 (43·9)	113 (58·5)	80 (41·5)
**Nodule size**^c^**, No (%), cm**			
< 2·00	145 (86·3)	12 (70·6)	133 (88·1)
2·00-4·00	21 (12·5)	4 (23·5)	17 (11·3)
> 4·00	2 (1·2)	1 (5·9)	1 (0·7)
**Nodule Lobe Position, No (%)**			
Left	175 (39·8)	83 (47·4)	92 (52·6)
Right	236 (53·6)	139 (58·9)	97 (41·1)
Isthmus	29 (6·6)	17 (58·6)	12 (41·4)

a Five patients with two nodules. One patient with both nodules with negative result. One patient with both nodules with positive result. Three patients with one nodule with negative and the other with a positive result.

b According to the origin pathology lab (real-world).

c Nodule sizes were retrieved only by the postsurgical AP reports. AUS/FLUS, atypia of undetermined significance/follicular lesion of undetermined significance; FN/SFN, follicular or oncocytic (Hürthle cell) neoplasm/suspicious for a follicular or oncocytic (Hürthle cell) neoplasm; No, number of.

Although the mir-THYpe test was negative for malignancy in 239 samples (Benign Call Rate [BCR] of 54·3%), 17 nodules (7·1%) underwent thyroidectomy (Group A), (nine previously classified as Bethesda III [52·9%], and eight as Bethesda IV [47·1%]). The mean age of these 17 patients was 49 years, the mean nodule size was 1·8 cm, and the mean test-to-surgery time was 170 days; for details, see Supplementary Table S1. The mean age of the other 222 patients (92·9%) (Group B) was 52 years. The mean follow-up time was 412·9 days. Of these, 117 nodules (52·7%) were classified as Bethesda III and 105 (47·3%) as Bethesda IV; Supplementary Table S2 presents the 95% confidence intervals and ranges. Follow-up times are described in Supplementary Table S3.

The mir-THYpe test was positive for malignancy in 201 samples (Positive Call Rate [PCR] of 45·7%), and 184 nodules (91·6%) were surgically treated (Group A). However, 25 nodules (13·6%) were excluded due to lack of access to the original postsurgical AP report, and eight samples (4·3%) were excluded because the punctured nodule described in the FNA cytology did not correlate with the nodule(s) described in the postsurgical AP report (all AP reports described at least one benign and one malignant nodule and limited information on nodule side, position and size were provided, making the exact correlation impossible). Of the remaining 151 resected nodules (82·1%), 83 were previously classified as Bethesda III (55%) and 68 as Bethesda IV (45%). The mean age of the patients was 49 years, the mean nodule size was 1·13 cm, and the mean test-to-surgery time was 79 days. The mean age of the other 17 patients (8·4%) (Group B) was 54 years, and the mean follow-up time was 359·6 days. Among them, 13 nodules (76·5%) were classified as Bethesda III and four (23·5%) as Bethesda IV; Supplementary Table S2 presents the 95% confidence intervals and ranges. Test-to-surgery and follow-up times are described with periods in Supplementary Table S3.

Overall, the “typical” profile of a real-world patient referred for the mir-THYpe test was women (82%), younger than 55 years (57·3%), with a Bethesda III (56·1%) thyroid nodule <2 cm (86·3%). Table 1 displays the demographic and clinical characteristics of the study cohort.

### Test performance

Table 2 summarises the test performance across all 168 resected nodules according to the histopathological type found post-surgery.

**Table 2:** Performance of microRNA-based molecular classifier according to histopathological subtype**.**

	FNA smear slides Bethesda classes		
mir-THYpe result / Postsurgical tissue	AUS/FLUS (III)	FN/SFN (IV)	Total
**Test-Negative nodules**	**9**	**8**	**17**
**Malignant -** False Negatives, No (%)	**0 (0%)**	**0 (0%)**	**0 (0%)**
**Benign** - True Negatives, No (%)	**9 (100%)**	**8 (100%)**	**17 (100%)**
Hürthle cell adenoma	2	2	4
Follicular Adenoma	1	3	4
Adenomatous goitre / follicular hyperplasia	-	2	2
Colloid goitre	5	1	6
Hashimoto's thyroiditis	1	-	1
**Test-Positive nodules**	**83**	**68**	**151**
**Malignant** - True Positives, No (%)	**52 (62·7)**	**48 (70·6)**	**100 (66·2%)**
Papillary carcinoma, usual type	23	7	30
Papillary carcinoma, follicular variant	16	30	46
Papillary carcinoma, Hürtle cell variant	2	1	3
NIFTP^a^	6	3	9
Follicular carcinoma, microinvasive	4	5	9
Follicular carcinoma, widely invasive	-	1	1
Follicular carcinoma, Hürtle cell variant	-	1	1
Medullary carcinoma	1	-	1
**Benign** - False Positives, No (%)	**31 (37·3)**	**20 (29·4)**	**51 (33·8%)**
Hürthle cell adenoma	4	3	7
Follicular Adenoma	10	11	21
Adenomatous goitre / follicular hyperplasia	9	5	14
Colloid goitre	6	1	7
Hashimoto's thyroiditis	2	-	2
**Total**	**92 (54·8%)**	**76 (45·2%)**	**168 (100%)**

a Considering positive test result for NIFTP as correct classification. NIFTP, Non-invasive follicular thyroid neoplasm with papillary-like nuclear features; AUS/FLUS, atypia of undetermined significance/follicular lesion of undetermined significance; FN/SFN, follicular or oncocytic (Hürthle cell) neoplasm/suspicious for a follicular or oncocytic (Hürthle cell) neoplasm; No, number of.

All 17 samples with negative results treated with surgery, regardless of Bethesda class, were confirmed as benign lesions (true negatives) in the postsurgical AP reports, achieving an overall NPV of 100% (one-side CI97·5% 80·5–100). This result must be interpreted with caution because the sample size was too small (patients with a negative test usually do not receive a surgery recommendation). This result included four Hürthle cell adenomas (23·5% [CI95% 6·8–49·9]).

Of the 151 resected nodules with positive results included in the final analysis, 100 nodules were confirmed as cancer/NIFTP lesions (true positives) in the postsurgical AP report, including nine NIFTPs (9% [CI95% 4·2–16·4]) and one medullary carcinoma (1% [CI95% 0·02–5·5]), achieving an overall positive predictive value (PPV) of 66·2% (CI95% 60·3–71·7). Splitting the results by cytological class when the mir-THYpe test was positive, 52 of the 83 Bethesda III (62·7%), and 48 of the 68 Bethesda IV nodules (70·6%) were accurately classified. Fifty-one nodules showed false-positive results due to benign tissue identified in the postsurgical AP reports (33.8% [CI95% 26·3–41·9]).

Overall, the test accuracy was 70% (CI95% 62–76·5) as it accurately classified 117 of the 168 resected samples.

Although expected, the small number of resected test-negative nodules impaired the realistic calculation of the sensitivity and NPV (both at 100%). To minimise this effect, we performed a theoretical calculation based on the Bayes’ theorem,^19^ applying the sensitivity observed during the validation study^13^ (94·6%) to the 222 non-resected nodules with test-negative results (Figure 1, box f), resulting in 210 true negatives and 12 false negatives. In this scenario (base case), the mir-THYpe test achieved 89·3% sensitivity (CI95% 82–94·3), 81·65% specificity (CI95% 76·6–86), 66·2% PPV (CI95% 60·3–71·7), and 95% NPV (CI95% 91·7–97) at a 28·7% (CI95% 24·3–33·5) disease prevalence (Table 3). Sensitivity analysis in other two scenarios, excluding the 17 test-negative resected-nodules (scenario #1) and analysing all the 239 test-negative nodules (regardless of the 17 test-negative resected-nodules surgery results) (scenario #2) demonstrated that the test NPV range from 81·6 to 99·2% at a 95% confidence interval, (See Supplementary Table S5).

**Table 3:** Calculated performance of microRNA-based molecular classifier in cytologically indeterminate thyroid nodules.

	Postsurgical tissue class, no		Test performance		
*Result*	Cancer+NIFTP	Benign	Analytical parameter	%	CI95%
**Positive**	100	51	**Sensitivity**	89·3	82-94·3
			**Specificity**	81·6	76·6–86
**Negative**	**12**^a^	**210**^a^ +17 **(227)**^a^	**NPV**	95·0	91·7–97
			**PPV**	66·2	60·3–71·7

a Theoretical values, considering the published sensitivity (13) of 94·6% for the 222 test-negative not surgically resected nodules. CI, Confidence Interval; NPV/PPV, Negative/Positive Predictive Value; NIFTP, Non-invasive follicular thyroid neoplasm with papillary-like nuclear features; No, number of.

### *Clinical decisions support and surgery reduction rates*

The mir-THYpe test supported 93% of clinical decisions when the result was negative for malignancy (222 nodules with no surgery, out of 239 test-negatives). Positive test results supported 91·5% of clinical decisions (184 nodules treated surgically, out of 201 test-positives). Overall, the mir-THYpe test supported 92·3% of clinical decisions (406/440 cases).

To calculate the rates of total avoided surgeries among the 440 eligible patients, we assumed that 423 would have undergone thyroidectomy if no molecular test were available (we subtracted 17 patients not treated surgically, despite a positive result – Figure 1, box k). Of these, 222 patients avoided surgery (Figure 1, box f), resulting in 52·5% (CI95% 47·6–57·3) of all surgeries.

Following Figure 1, we considered that 280 patients (17 [box e] + 222 [box f] + 51 [box o]) had a benign nodule and would undergo a “potentially unnecessary” surgery if no molecular test were available. As we could not assume that all 222 samples from box f were truly benign, based on the Bayes’ theorem,^19^ we applied the sensitivity observed in the validation study^13^ (94·6%) and estimated that around 210 samples were true-negative nodules in this cohort, resulting in a total of 268 patients who would have undergone a “potentially unnecessary” surgery if no molecular test were available. Considering that only 68 patients (17 [box e] + 51 [box o]) were surgically treated, the mir-THYpe test avoided 74·6% (CI95% 69–79·2) of “potentially unnecessary” surgeries.

## Discussion

The present study aimed to prospectively evaluate the real-world performance, the support on clinical decisions, and the true potential of a previously described microRNA-based thyroid molecular classifier for avoiding surgeries in a multicentre cohort.^13^

Although randomised controlled trials (RCTs) are considered the gold standard for analysing the efficacy of therapies, in order to associate cause and effect due to control groups, they can be limited to a subset of patients who could not be fully representative of the unselected real-world patients.^22^, ^23^, ^24^ Real-world evidence studies might better represent routine practice, providing a valuable reflection on the range and distribution of patients observed in clinical practice and including the bias of mis-classification due to physician judgement during clinical care.^23^, ^24^, ^25^ A real-world evidence study design can tell physicians which results they might experience in practice, providing a clear view of which level of support in clinical decisions the molecular tests can provide.

The analysed specimens belonged to patients for whom a physician considered the molecular testing a clinical indication in real-world, thus avoiding the bias of including retrospective samples based on the Bethesda classification only. Another advantage was that the FNA smear slides were sent at room-temperature. The FNA smear slides were prepared at 128 cytopathology laboratories, ensuring a highly heterogeneous sample cohort. As summarised in Table 1, patient demographics and nodule characteristics corroborated the expected real-world proportions, as illustrated in the “typical” patient profile described above. Lastly, patient and physician received the molecular test results before deciding the next step (unlike blinded studies in which all nodules are resected, and test support in clinical decisions is unmeasurable). Therefore, we could follow the patients and observe the real impact of their more-informed clinical decisions and test utility on surgery avoidance. The support of the mir-THYpe test on real-world clinical decision-making (92·3% overall) re-emphasises that the Bethesda categories III and IV present a challenge for physicians to decide the best option for their patients, with 74·5% of “potentially unnecessary” surgeries being avoided, suggesting that the molecular test could minimise this problem and potentially improve the efficiency of the healthcare system.

However, our study had limitations. Due to the real-world study design, we were not able to collect important pre-test nodule risk characteristics, such as ultrasonographic characteristics of the nodules, and, in Bethesda III cases, to identify whether the tested sample was from the first FNA or from the repeat FNA. The lack of these pre-test data limited our conclusions for what nodule risk characteristics might have been preferentially selected by physicians during clinical practice. The multicentre design made it difficult to closely monitor patients as they were not centralised to a few institutions, and there was limited access to other clinical parameters. Ideally, non-operated patients should be followed with US at least to identify possible nodule progression suggestive of management changes. Although the physicians routinely monitored the patients, we had restricted access. Therefore, we had to limit the follow-up monitoring according to whether the patient underwent thyroid surgery or not and we could not access the reasons why the 17 test-negative patients underwent surgery and the 17 test-positive patients were not surgically treated (clinical decision or patients’ choice). We were also unable to collect nodule size of the non-operated patients, limiting the understanding of the profile of patients undergoing the test in real-world. Moreover, we did not review the patients’ FNA or AP slides in order to guarantee the real-world approach, even though we assumed the risk of potential misclassifications at the pathology laboratories. Finally, as most patients who tested negative did not undergo thyroid surgery (222/239, 93%), we did not have the statistical power to evaluate the performance of negative results (mainly sensitivity and NPV). Although all 17 resected nodules with negative results were confirmed benign, the sample size impaired the confidence on 100% sensitivity and NPV. However, the statistical adjustment based on Bayes’ theorem applied (described above on Results) for the 222 test-negative samples to minimise this effect and calculate the test performance was valid as the sensitivity of a diagnostic test is a parameter not influenced by disease prevalence. Thus, in this specific and supposed scenario, the present study achieved an NPV of 95%, which seems realistic (similar to the NPV of 95·9% observed in the previous validation study).^13^ The sensitivity analysis of the data in other two supposed scenarios (See Supplementary Table S5) also suggests a high NPV of the mir-THYpe test in real-world, but not at 100%, as observed in the small cohort of 17 test-negative resected nodules, all with benign histology.

The statistical parameters used to analyse the performance of a test are crucial in determining practical applications and clinical adoption. To evaluate the real-world performance of the mir-THYpe test, we contextualised it with the results of prospective studies on the latest version of two leading commercially available thyroid molecular classifier tests, the ThyroSeq v3^20^ and Afirma GSC.^21^ We also compared the molecular tests results with the performance of the FNA alone using data from a meta-analysis.^10^

ThyroSeq v3 and Afirma GSC are the two main thyroid molecular classifiers available, widely adopted worldwide with proven clinical impact, but restricted in poor countries due to price and logistics. Hence, we compared the mir-THYpe test performance observed in this study with prospective studies describing the performance of the latest version of these two tests.^20^^,^^21^ Based on the true positives for each test, the mir-THYpe achieved similar performance to the ThyroSeq v3 test (62·7% vs. 64%, 70·6% vs. 68·1%, and 66·2% vs. 66% for Bethesda III, IV, and overall, respectively) and better performance than the Afirma GSC in all scenarios (62·7% vs. 44·7%, 70·6% vs. 47·4%, and 66·2% vs. 45·8% for Bethesda III, IV, and overall). In practical and quantitative terms, when either mir-THYpe or ThyroSeq v3 is used alongside FNA indeterminate cytology, around 1·5 surgeries are required to identify one cancer patient. If Afirma GSC is chosen, 2·18 surgeries are required to identify one cancer patient (1·45 more than when using mir-THYpe) and 4·43 surgeries are required with FNA alone (2·94 more than adding mir-THYpe) (See Supplementary Table S4). Based on the NPV of each test, the mir-THYpe achieved similar performance (95%) to ThyroSeq v3 (97%) and Afirma GSC (96%). Moreover, the sample type requested to perform the test is also relevant, because if no material was stored from the first FNA (frozen), ThyroSeq v3 and Afirma GSC need a new and dedicated FNA. Furthermore, delicate logistics operations are needed as samples must be refrigerated and delivered on time. Although ThyroSeq v3 is under validation for FNA smear slides, the available results are limited to only 31 samples, with different performance depending on slide staining (Papanicolaou, 100%; Diff-Quick, 65%; and overall, 81%).^26^ Furthermore, FNA smear slides were not used for the prospective study validation.^20^

## Conclusion

The reported data demonstrate over a heterogeneous and large cohort of patients that the real-world use of the mir-THYpe test can reduce surgery rates for Bethesda III and IV indeterminate cytology nodules and support clinical decisions. Moreover, the real-world test performance is robust and comparable to other well-established thyroid molecular classifiers. Furthermore, it can be performed using already available cytology smear slides at a significantly lower cost. Cost-effectiveness analyses of the test's financial impact and healthcare system savings are under development, and together with the present data, will help guide new policies.

## Contributors

MTS designed and supervised the study. CPC, DLAF, GMM, RAS, CF, GFR, RC, HER, FV and MV contributed with data and medical expertise. MTS, BMR, SS, AFO and MO contributed with data collection, verification, integration, and patients' follow-up. MTS drafted the manuscript. MTS and BMR contributed as molecular biologists. CPC contributed with statistical analysis. All authors read and approved the final version.

## Data sharing statement

Due to the nature of this research, to protect the participants’ privacy and confidentiality, raw data are not shared publicly. Relevant data supporting the findings and conclusions of this study are available within the article and/or supplementary materials. Upon a justifiable request, the share of de-identified data should be approved by the board of an investigational ethics committee.

## Declaration of interests

MTS holds equity at Onkos Molecular Diagnostics. BMR, SS, AFO and MO are formal employees at Onkos Molecular Diagnostics. All other authors declare no competing interests.

## Appendix Supplementary materials

Image, application 1
