# Supplementary material for: Clinical decision support analysis of a microRNA-based thyroid molecular classifier: A real-world, prospective and multicentre validation study
Source: eBioMedicine. 2022 Jul 1;82:104137. doi: 10.1016/j.ebiom.2022.104137 (PMC9254359; doi:10.1016/j.ebiom.2022.104137)
Supplement: Supplementary file 1 [file mmc1.docx]

**SUPPLEMENTARY MATERIAL**

*Clinical Decision Support analysis of a microRNA-based Thyroid Molecular Classifier: A Real-world, Prospective and Multicentre validation study*

**
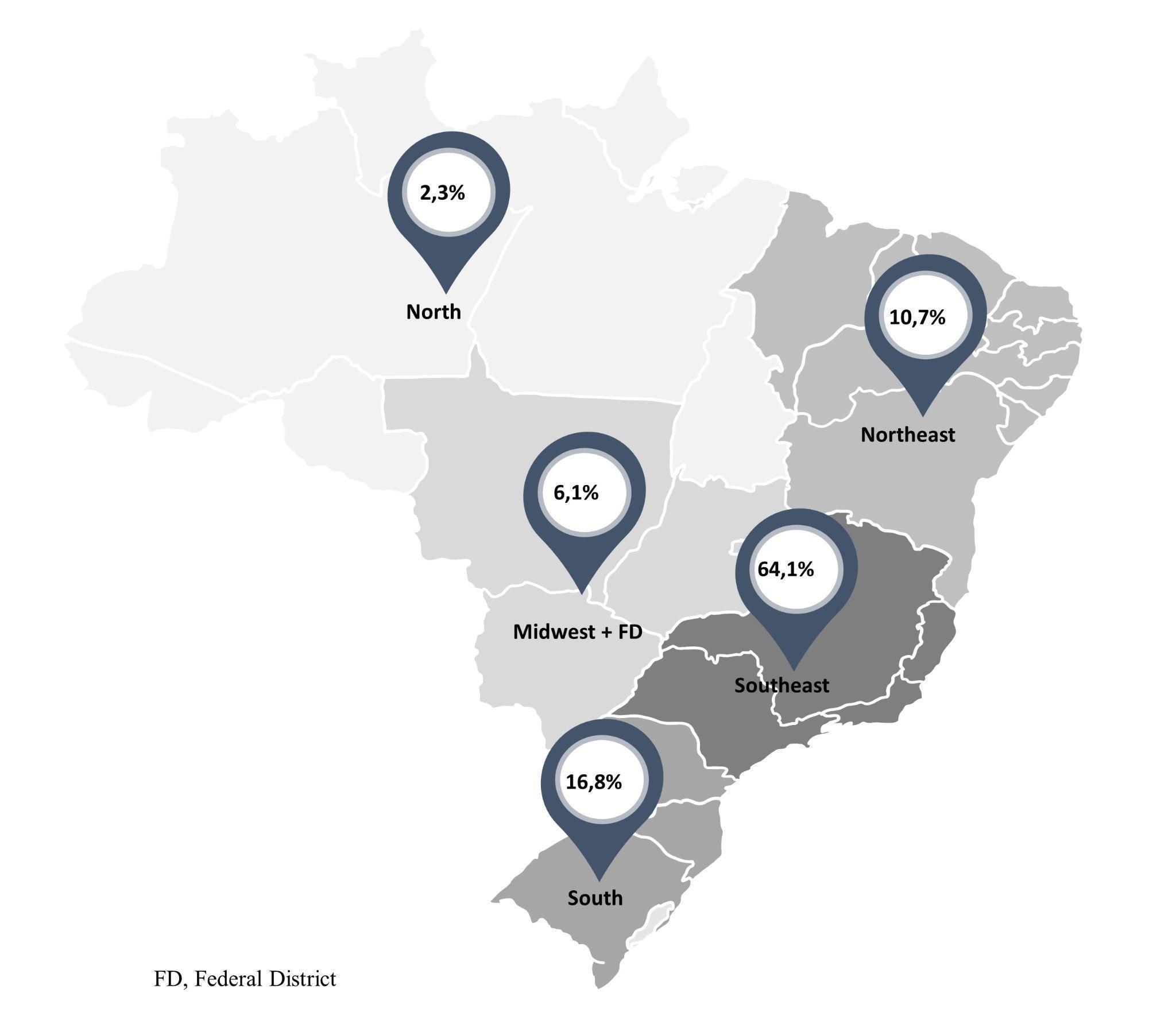
**

**Figure S1. Geographic distribution of the 128 cytopathology labs where the 440 FNA smear slides tested in the study were prepared and the Bethesda categories assigned.**

**Table S1.** **Clinical details of the 17 patients with a test-negative result with nodules surgically resected (all true negatives).**

| Patient | Bethesda class | Postsurgical histological subtypes | Test-to surgery (days) | Nodule size (cm) | Nodule lobe position | Sex |
| --- | --- | --- | --- | --- | --- | --- |
| 1 | III | Colloid goitre | 25 | 1·5 | Right | Female |
| 2 | III | Colloid goitre | 39 | 0·5 | Right | Male |
| 3 | III | Colloid goitre | 142 | 1 | Left | Female |
| 4 | III | Colloid goitre | 350 | 1·5 | Left | Female |
| 5 | III | Colloid goitre | 412 | 1·4 | Right | Female |
| 6 | III | Follicular Adenoma | 76 | 3 | Left | Female |
| 7 | III | Hashimoto’s thyroiditis | 76 | 2 | Isthmus | Female |
| 8 | III | Hürthle cell adenoma | 78 | 5·3 | Right | Male |
| 9 | III | Hürthle cell adenoma | 182 | 3 | Right | Female |
| 10 | IV | Adenomatous goitre / follicular hyperplasia | 74 | 1 | Isthmus | Female |
| 11 | IV | Adenomatous goitre / follicular hyperplasia | 320 | 1·7 | Left | Male |
| 12 | IV | Colloid goitre | 115 | 1·5 | Isthmus | Female |
| 13 | IV | Follicular Adenoma | 52 | 1·5 | Right | Female |
| 14 | IV | Follicular Adenoma | 120 | 1·4 | Left | Female |
| 15 | IV | Follicular Adenoma | 321 | 3 | Left | Male |
| 16 | IV | Hürthle cell adenoma | 117 | 0·7 | Isthmus | Male |
| 17 | IV | Hürthle cell adenoma | 390 | 0·6 | Right | Female |
|  |  | *Mean* | *169·9* | *2·2* |  |  |
|  |  | *CI95%* | *101·8–238·19* | *1·19–2·41* |  |  |

CI, Confidence Intervals; Ages, range 22–70; mean 49 years (CI95% 41·1–56·8). Ages are not specified to each patient to preserve patient’s confidentiality and privacy

**Table S2.** **Detailed Demographic and Clinical characteristics of the study by cohort groups.**

|  | mir-THYpe test result | |
| --- | --- | --- |
|  | **Negative** | **Positive** |
| Total | 239 (54·3) [49·5–59] | 201 (45·7) [40·9–50·5] |
| Group A (Surgery) |  |  |
| Nodules resected, No (%) [CI95%] | 17 (7·1) [4·2–11·1] | 184 (91·6) [86·8–95] |
| Included, No (%) [CI95%] | 17 (100) [80·5–100^a^] | 151 (82·1) [75·7–87·3] |
| *Bethesda III, No (%) [CI95%]* | *9 (52·9) [27·8–77]* | *83 (55) [46·7–63]* |
| *Bethesda IV, No (%) [CI95%]* | *8 (47·1) [23–72·2]* | *68 (45) [36·9–53·3]* |
| Corrected classified, No (%) [CI95%] | 17 (100) [80·5–100^a^] | 100 (66·2) [58·1–73·7] |
|  |  |  |
| Age, mean (range) [CI95%] years | 49 (22–70) [41·1–56·8] | 49 (22–85) [46·8–51·2] |
| Nodule Size, mean (range) [CI95%] cm | 1·8 (0·5–5·3) [1·2–2·4] | 1·13 (0·05–4·2) [1·02–1·24] |
| Test-to-surgery, mean (range) [CI95%] days | 170 (25–412) [101·8–238·2] | 79 (16–326) [68·9–89] |
|  |  |  |
| Group B (No-Surgery) |  |  |
| Nodules followed-up, No (%) [CI95%] | 222 (92·9) [88·7–95·8] | 17 (8·4) [5–13·2] |
| *Bethesda III, No (%) [CI95%]* | *117 (52·7) [45·9–59·4]* | *13 (76·5) [50·1–93·2]* |
| *Bethesda IV, No (%) [CI95%]* | *105 (47·3) [40·6–54·1]* | *4 (23·5) [6·8–49·9]* |
|  |  |  |
| Age, mean (range) [CI95%] years | 52 (20–86) [50·2–53·8] | 54 (29–90) [44·7–63·2] |
| Follow-up time, mean (range) [CI95%] days | 412·9 (120–796) [388·2–437·4] | 359·6 (120–733) [266·5–452·6] |

^a^ CI97%, one side

CI, Confidence Intervals; No, number of

**Table S3. Test-to-surgery and follow-up periods of the study by cohort groups.**

|  | mir-THYpe test result | | |
| --- | --- | --- | --- |
| *Period, No (%) days* | **Negative** | | **Positive** |
| Test-to-surgery (Group A - Surgery) | **17 (7·1)** | | **151 (89·9)** |
| < 30 | 1 (5·9) | | 21 (13·9) |
| 31–60 | 2 (11·8) | | 59 (39·1) |
| 61–90 | 4 (23·5) | | 28 (18·5) |
| 90–120 | 3 (17·6) | | 12 (7·9) |
| 121–150 | 1 (5·9) | | 16 (10·6) |
| 151–300 | 1 (5·9) | | 13 (8·6) |
| > 300 | 5 (29·4) | | 2 (1·3) |
|  |  | |  |
| Follow-up (Group B - No-surgery) | **222 (92·9)** | | **17 (10·1)** |
| 120–180 | 32 (14·4) | | 4 (23·5) |
| 181–360 | 63 (28·4) | | 4 (23·5) |
| 361–540 | 65 (29·3) | | 7 (41·2) |
| 541–720 | 45 (20·3) | | 1 (5·9) |
| 720–796 | 17 (7·7) | | 1 (5·9) |
|  | |  |  |
| *Total* | | **239 (100)** | **168 (100)** |

No, number of

**Table S4. Calculated number of surgeries needed to find 1 cancer case of the molecular tests and of the FNA alone according to the published of each study.**

|  | **Surgeries needed to find 1 cancer case (rates)** | | | **Differences compared to**  **the mir-THYpe (%)** |
| --- | --- | --- | --- | --- |
| *Tests / Bethesda Class* | **AUS/FLUS (III)** | **FN/SFN (IV)** | **III and IV** |  |
| **mir-THYpe** | 1·60 | 1·42 | 1·51 | na |
| **ThyroSeq v3**^1^ | 1·56 | 1·47 | 1·52 | 0 |
| **Afirma GSC**^2^ | 2·24 | 2·11 | 2·18 | +45% |
| **FNA alone**^3^ | 6·30 | 3·83 | 4·43 | +194% |

AUS/FLUS, atypia of undetermined significance/follicular lesion of undetermined significance; FN/SFN, follicular or oncocytic (Hürthle cell) neoplasm/suspicious for a follicular or oncocytic (Hürthle cell) neoplasm; na, not applicable.

Rates, 100 / % true positive

**Table S5. Sensitivity analysis of test performance considering different scenarios for the test-negative nodules.**

|  |  |  |  |  |  |  |  |  |  |
| --- | --- | --- | --- | --- | --- | --- | --- | --- | --- |
|  | **Base Case** | | | **Scenario #1** | | | **Scenario #2** | | |
|  | **Lower Limit** | **Central** | **Upper Limit** | **Lower Limit** | **Central** | **Upper Limit** | **Lower Limit** | **Central** | **Upper Limit** |
| *n of cases* | 81·8% | 94·6% | 99·3% | 81·8% | 94·6% | 99·3% | 81·8% | 94·6% | 99·3% |
| **True Positive** | 100 | 100 | 100 | 100 | 100 | 100 | 100 | 100 | 100 |
| **False Positive** | 51 | 51 | 51 | 51 | 51 | 51 | 51 | 51 | 51 |
| **False Negative** | 40 | 12 | 2 | 40 | 12 | 2 | 44 | 13 | 2 |
| **True Negative** | 182+17 (199) | 210+17 (227) | 220+17 (237) | 182 | 210 | 220 | 195 | 226 | 237 |
|  |  |  |  |  |  |  |  |  |  |
| *Parameter % (CI95%)* |  | | |  | | |  | | |
| **Sensitivity** | 71·7% (63–79) | 89·3% (82–94) | 98% (93–99) | 71·4% (63–79) | 89·3% (82–94) | 98% (93–100) | 69·4% (61–77) | 88·5% (81–94) | 98% (93–99) |
| **Specificity** | 79·6% (74–84) | 81·6% (77–86) | 82·3% (77–87) | 78·1% (72–83) | 80·4% (75–85) | 81·2% (76–86) | 79·2% (74–84) | 81·6% (77–86) | 82·3% (77-87) |
| **NPV** | 83·3% (79–87) | 95% (92–97) | 99·2% (97–100) | 82% (77–86) | 94·6% (91–97) | 99·1% (97–100) | 81·6% (77–85) | 94·6% (91–97) | 99·2% (97–100) |
| **PPV** | 66·2% (60–72) | 66·2% (60–72%) | 66·2% (60–72) | 66·2% (60–72) | 66·2% (60–72%) | 66·2% (60–72) | 66·2% (60–72) | 66·2% (60–72%) | 66·2% (60–72) |

Considering the previously published sensitivity (4) of 94·6% (CI95% 81·8–99·3).

Base Case: Previously published sensitivity applied to the 222 test-negative non-resected-nodules + 17 test-negative, resected-nodules

Scenario #1: Previously published sensitivity applied to the 222 test-negative non-resected-nodules excluding the 17 test-negative, resected-nodules due to potential bias

Scenario #2: Previously published sensitivity applied to the 239 test-negative nodules, regardless surgery status

**References**

1. Steward DL, Carty SE, Sippel RS, et al. Performance of a multigene genomic classifier in thyroid nodules with indeterminate cytology: a prospective blinded multicenter study. *JAMA Oncol*. 2019;**5**:204–212. doi:10.1001/jamaoncol.2018.4616
2. Patel KN, Angell TE, Babiarz J, et al. Performance of a genomic sequencing classifier for the preoperative diagnosis of cytologically indeterminate thyroid nodules. *JAMA Surg*. 2018;**153**:817–824. doi:10.1001/jamasurg.2018.1153
3. Bongiovanni M, Spitale A, Faquin WC, et al. The Bethesda System for Reporting Thyroid Cytopathology: a meta-analysis. *Acta Cytol*. 2012;**56**:333–339. doi:10.1159/000339959
4. Santos MT, Buzolin AL, Gama RR, et al. Molecular classification of thyroid nodules with indeterminate cytology: development and validation of a highly sensitive and specific new miRNA-based classifier test using fine-needle aspiration smear slides. *Thyroid*. 2018; **28**:1618–1626
